# Characterisation of the LMS200 Laser Beam under the Influence of Blockage Surfaces. Influence on 3D Scanning of Tree Orchards

**DOI:** 10.3390/s110302751

**Published:** 2011-03-02

**Authors:** Ricardo Sanz-Cortiella, Jordi Llorens-Calveras, Joan R. Rosell-Polo, Eduard Gregorio-Lopez, Jordi Palacin-Roca

**Affiliations:** 1 Department of Agro-Forestry Engineering, University of Lleida, Avinguda Rovira Roure 191, 25198 Lleida, Spain; E-Mail: jr.rosell@eagrof.udl.cat; 2 Department of Agri Food Engineering and Biotechnology, Polytechnic University of Catalonia, Campus del Baix Llobregat, Edifici D4, Av. del Canal Olímpic, s/n 08860 Castelldefels, Spain; E-Mail: jordi.llorens.calveras@upc.edu; 3 Department of Agro-Forestry Engineering, University of Lleida, Edifici CREA, C/Pere de Cabrera s/n 25001, Lleida, Spain; E-Mail: egregorio@eagrof.udl.cat; 4 Department of Computer Science and Industrial Engineering, University of Lleida, Av. Jaume II 69, 25197 Lleida, Spain; E-Mail: palacin@diei.udl.cat

**Keywords:** mixed pixels, terrestrial LIDAR, laser measurement, 3D plant structure

## Abstract

The geometric characterisation of tree orchards is a high-precision activity comprising the accurate measurement and knowledge of the geometry and structure of the trees. Different types of sensors can be used to perform this characterisation. In this work a terrestrial LIDAR sensor (SICK LMS200) whose emission source was a 905-nm pulsed laser diode was used. Given the known dimensions of the laser beam cross-section (with diameters ranging from 12 mm at the point of emission to 47.2 mm at a distance of 8 m), and the known dimensions of the elements that make up the crops under study (flowers, leaves, fruits, branches, trunks), it was anticipated that, for much of the time, the laser beam would only partially hit a foreground target/object, with the consequent problem of mixed pixels or edge effects. Understanding what happens in such situations was the principal objective of this work. With this in mind, a series of tests were set up to determine the geometry of the emitted beam and to determine the response of the sensor to different beam blockage scenarios. The main conclusions that were drawn from the results obtained were: (i) in a partial beam blockage scenario, the distance value given by the sensor depends more on the blocked radiant power than on the blocked surface area; (ii) there is an area that influences the measurements obtained that is dependent on the percentage of blockage and which ranges from 1.5 to 2.5 m with respect to the foreground target/object. If the laser beam impacts on a second target/object located within this range, this will affect the measurement given by the sensor. To interpret the information obtained from the point clouds provided by the LIDAR sensors, such as the volume occupied and the enclosing area, it is necessary to know the resolution and the process for obtaining this mesh of points and also to be aware of the problem associated with mixed pixels.

## 1. Introduction

As a result of a series of different factors, agricultural work is finding that it requires the use of ever higher levels of sophisticated technology. These factors include: an ever increasing demand for quality and quantity from agricultural goods; the limitations of currently available resources; enormous worldwide competition; a growing sensitivity to environmental questions; and greater legislative pressure. In response to the above, there has been a growing interest in “Precision Agriculture”. This basically entails using fewer agricultural inputs (energy, water, fertilizers, seeds, plant protection products, *etc.*) and improving the application of techniques to supply the necessary dosages at the most appropriate times and locations. This offers savings in costs, increased production, improvements in quality and reductions in environmental damage. New information technologies, like the Internet, Geographic Information Systems (GIS), the evolution of electronics, and the appearance of new, faster and more reliable sensors with new capabilities, mean that the concept of precision agriculture has now become more of a practical reality.

In the field of fruit cultivation, determining the positions of trunks, branches, leaves, flowers and fruits and quantifying them are necessary prerequisites to any work to be undertaken on trees. The geometric characterisation of orchards is a non-destructive precision activity which entails measuring and acquiring precise knowledge of the geometry and structure of trees. This is both an important and complex task to perform. It is important because a whole series of agricultural activities, including phytosanitary treatments, irrigation, fertilization and pruning, largely depend on the structural and geometric properties of the visible part of trees. It is complex because of the numerous elements which make up trees and the difficulties associated with measuring them. There are three basic reasons for this difficulty: (i) the large number of elements involved; (ii) the layout of a relatively small, three-dimensional, space which—from any viewpoint—will always have elements that are totally or partially hidden, and (iii) the geometric complexity of all the elements.

The measurement and structural characterisation of plants can be carried out remotely by several detection approaches, such as image analysis techniques, stereoscopic photography, analysis of the light spectrum, ultrasonic sensors, light detection, and ranging (LIDAR) laser sensors [1]. Image-based canopy measurements require elaborate algorithms and fast computational resources in order to operate in real-time. The angle of divergence of the ultrasonic waves limits the spatial resolution and accuracy of the ultrasonic sensors.

LIDAR is a remote laser range sensor based on measurement of the time lapse between the transmission of a pulsed laser beam and the reception of its echo from a reflecting object (target); this time-of-flight (TOF) is used to estimate the distance between the laser and the target. These sensors are normally able to measure thousands of distances per second with great accuracy. Their main use in precision fruit growing is to provide three-dimensional(x, y, z) point clouds which, with the use of appropriate algorithms, allow the structure of the trees to be described and reconstructed with a very high degree of accuracy [2–5].

The SICK LMS200 LIDAR land sensor was chosen to carry out the work described in this paper. The main reasons for this selection were:

1. This is an internationally known sensor which is widely used within the industry for such varied applications as: (i) surveillance systems; (ii) systems for counting and measuring objects; (iii) anti-collision systems; iv) vision systems for robots and self-guided equipment [6], *etc.*
2. It is a 2D sensor that only scans in one plane. This makes its cost very low compared to 3D LIDAR land sensors which generally make more precise sweeps of three-dimensional spaces, and with a greater range of distance than the LMS200. These 3D sensors are mainly designed for very precise topographical applications.
3. The technical specifications described in Section 2.1 make it appropriate for use in the geometric characterisation of orchard crops.
4. In recent years, this method has begun to be increasingly used in both forestry [7,8] and agriculture [9,10]. This sensor is now being used for the geometric characterisation of a variety of crops including apple, pear, peach, vine and citrus [1,5,11,12].

When a laser spot is located at the very edge of a target, the measured range corresponds to a combination of the foreground and background targets, *i.e.*, the range falls between the distances to the foreground and background targets. The name given to this range is “mixed pixels” [13].

Given the known dimensions of the laser beam cross-section of the LMS200 sensor as provided by the manufacturer (Table 1), and given the known dimensions of the elements which make up the elements to be studied, it is anticipated that the laser beam will very often not fully impact on a single target (flower, leaf, fruit, branch, trunk), but that it will rather impact on two or more elements located at different distances from the sensor. Knowing what is happening in these situations is the main objective of this study. With this purpose in mind the specific aims are as follows: (i) to establish a visualisation method of the laser beam which will enable a good description of it; (ii) to determine the geometry of the beam and (iii), to characterise the performance of the distance measurement when the laser beam falls on two targets/objects at different distances (mixed pixels).

A precedent to this work can be found in the studies undertaken by Ye and Borenstein [13], who used an LMS200 sensor to force the appearance of mixed pixels. That test involved making the laser beam partially fall on a first (foreground) target located 1 m from the sensor and the rest of the beam falling on a second (background) target located 2 m from the sensor. The study undertaken in this paper does not investigate this particular topic in any greater depth. Ye and Borenstein themselves explain [14], based on communication with the manufacturer Sick Germany, that the LMS200 sensor is a single shot measurement system which sends out a pulse with a width (ΔL) of approximately 1 m and detects reflection with this same pulse width. In corner shots, this detection scheme allows the receiver to receive reflections from both the edge and background (within a one metre pulse width length). When the distance (ΔD) between the edge of the foreground object and the background is close to the width of the laser pulse (ΔL ≈ 1 m for the Sick LRF), a substantial number of mixed pixels are generated. However, if ΔD > ΔL, the number of mixed pixels generated drops significantly. This is because the Sick LRF is designed to only accept reflections stemming from the pulse itself as valid readings. This smart design feature is very effective for rejecting most ambient noise, but it does not eliminate all of it. The work undertaken in the present study aimed to either verify or refute these affirmations and to look into the problem of mixed pixels associated with the LMS200 sensor in greater depth.

## 2. Materials and Methods

This section contains four parts. The Section 2.1 relates to the most relevant technical specifications of the LMS200 sensor. The Section 2.2 focuses on the visualisation and geometric characterisation of the laser beam emitted by the sensor, with the aim of finding new characteristics or properties not previously specified by the manufacturer. The Section 2.3 describes a trial in which the beam emitted by the sensor was partially obstructed by templates with different formats, at a fixed distance of 5 m. The part of the beam that was not obstructed then impacted on a second object located 30 cm behind the first. The objective of this test was to study the phenomenon of mixed pixels by varying the shape of the templates and the percentage and trajectory of their obstructive effects. The Section 2.4 describes another trial which continued the study of mixed pixels and in which the shape of the template and the obstruction trajectory were held constant, while the distance to the first obstructing object (*D_1_*), the obstruction percentage (*P_0_*), and the distance between the first and second objects were gradually modified (*D_en_*). The aim of this last test was to discover the function that relates these last three variables to the distance from the LMS200 sensor (*D_láser_*).

### 2.1. Description and Operation of the LMS200 Sensor

LIDAR sensors use one of two technologies to measure distance: phase comparison and the time-of-flight of the laser beam. The LMS200 uses this second type of technology. The emitted laser radiation is in the near infrared range and is therefore not visible. It has a wavelength of 905 nm. The laser has Protection Class 1 classification, meaning it is safe to view. This type of sensor is used to remotely measure distances at high frequency without any physical contact with the measured target. The number of measurements made per second is in the order of thousands. Targets with specific reflecting characteristics are not necessary and no other lighting is required than that provided by the emitted laser beam [15].

The LMS200 can operate in three different modes:

- Distance measurement.
- Measurement of received radiant power values.
- A combination of the first two options, obtaining distances and radiant power values in an angular range restricted to 100°.

The radiant power values obtained depend on three variables: the distance at which the target/object receives the impact from the laser beam; the optical properties of the target/object surface and; in the case of partial impacts, the proportion of the cross-section of the beam which falls on the target/object. In agricultural applications, we find all of the above cases and this makes interpretation of these values extremely difficult.

**Figure 1.**
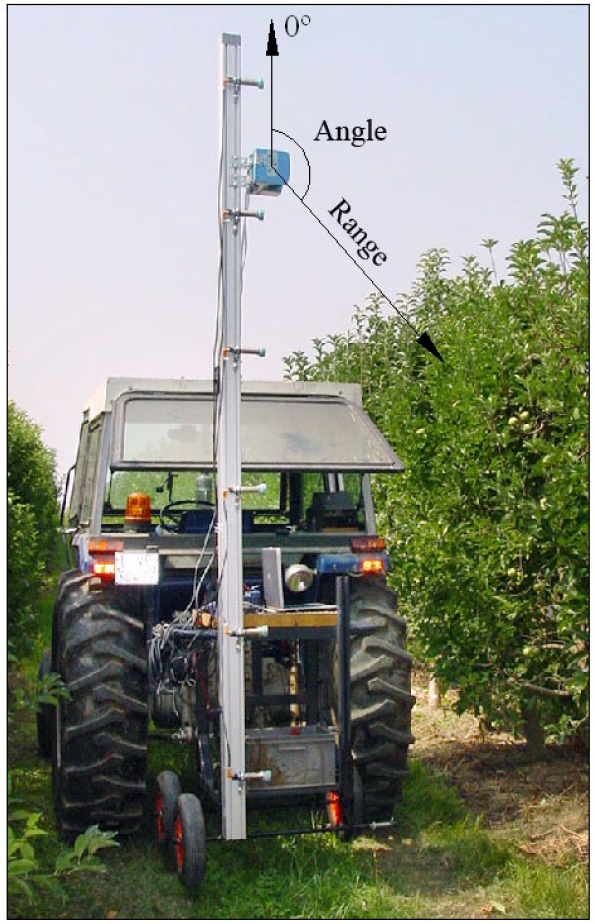
The impact points of the0 laser beam are determined by polar coordinates: range and angle.

The maximum angular range of coverage is 180° and smaller ranges can be configured if so desired. The angular resolution (angle of separation between contiguous laser beam paths) can be configured by the user with a choice of three possible values: 1°, 0.5° and 0.25°. The sensor gives the measures in polar form, providing the distance and angle (Figure 1). Within the range from 0 to 8 m, the measurement resolution is 1 mm and the standard deviation is ±15 mm. Data transfer between the computer and sensor is through serial port RS232 at speeds of 9,600, 19,600 or 38,400 bits per second or through serial port RS422 at a speed of 500,000 bits per second. The protection index of the sensor is IP65, which is sufficient for experiments. These technical characteristics mean that the LMS200 sensor is, in principal, suitable for the geometric characterisation of orchards

### 2.2. Visualization and Geometric Characterisation of the Laser Beam of the LMS200 Sensor

**Figure 2.**
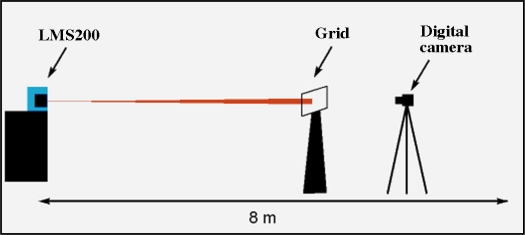
Set up for visualisation of the laser beam.

**Figure 3.**
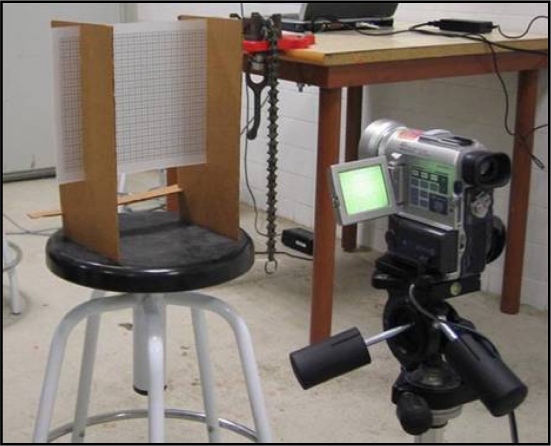
Plastic translucent grid template and digital camera used in characterisation of the laser beam.

The LMS200 emits a laser beam that is invisible to the naked eye. To visualise the beam, it was necessary to use a digital camera sensitive to near infrared light. The aim was to obtain detailed information about the beam that had not been provided by the manufacturer [15,16]. The system was set up as shown in Figures 2 and 3.

Once the beam had been visualised it was possible to geometrically characterise it. The visualisation procedure consisted of projecting a beam onto a translucent plastic grid template (Figure 3). The printed grid on the template permitted exact measurements from the photographs taken of the beam (see Figures 9, 10 in Section 3.1). The smallest subdivisions were 1 mm.

The digital camera used was a SONY DCR PC100 (Figure 3) with photographic capability. The infrared view mode called “Night Shot” was used to view the beam. The photographs obtained were stored in .jpg format with a resolution of 1,152 × 864 pixels.

### 2.3. First Mixed Pixel Test: Partial Blockage of the Laser Beam at a Distance of 5 m

**Figure 4.**
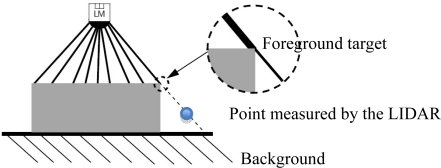
The edge effect produced when a beam partially falls on the edge of a target [16]. In this case the LIDAR sensor provides an intermediate range between the foreground and background objects.

The LMS200 laser sensor emits a laser beam with considerable divergence (measured in mrad). When this falls on targets located at standard distances (e.g., 1–8 m), it generates large spots (Table 1). When the sensor was used in fruit plantations in field work, it was common for part of the laser beam to fall on a foreground element (leaf, flower, fruit, branch, trunk) while the rest fell on background elements. The distances transmitted to the computer by the sensor were therefore the result of obtaining an initial distance value which underwent two internal corrections based on distance and reflectivity tables. In this process, it is generally assumed that the full beam falls on the desired target. In cases in which the laser beam does not fully fall on a single target (Figure 4), the internal corrections are not totally correct and, as a consequence, measurements of the distance emitted by the sensor do not exactly correspond to those associated with the foreground target [16]. In this case, the LIDAR sensor provides a measurement that lies between the distances to the foreground and background elements.

**Figure 5.**
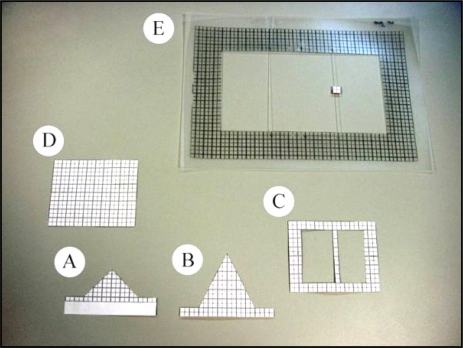
Shutter templates: **(A)** Triangle A. **(B)** Triangle B. **(C)** 5 mm wide rectangular strip. **(D)** Rectangle (10 × 8 cm). **(E)** Template frame and 1 cm^2^ template.

This study of the beam was undertaken considering the previously cited problems. The aim was to determine the reliability of the range value in different measurement situations. To study the behaviour of a given range value when it falls on two targets at different distances, the first tests were performed with partial obstruction of the laser beam at a range of 5 m using paper templates with different geometric shapes (shutter templates). The target which received the impact of the unblocked part of the beam (the grid template) was positioned at a distance of 30 cm from the foreground target. The 5 m distance was chosen simply because it was an intermediate distance between 0 and 8 m, the distance range configured in the sensor. The 30 cm separation between the foreground and background targets was chosen bearing in mind the use of the sensor in the field; it corresponded to the probable separation between two crop elements (flowers, fruits, leaves, branches and trunk). The test set-up (shown in Figure 7 in Section 2.4) was used to partially block the beam. Different types of shutter templates were positioned between the sensor and the plastic grid template (Figure 5). The two triangular-shaped templates were approximations to the shape of tree leaves. The frame of the templates [Figure 5(e)] contained two nylon threads over which the shutter templates were moved. In this way, it was possible to gradually block the beam, enabling us to observe the evolution of the measurements taken by the sensor. Photographs taken with a digital camera and processed by the 2004 version of AUTOCAD (Autodesk, Inc.) software were used to measure the exact blocked surface area of the beam.

### 2.4. Second Mixed Pixel Test: Partial Blockage within the Range from 0 to 8 m

In the tests, it was observed that the distance measurements obtained when the laser beam fell on two targets at different distances depended on at least the following variables:

- D_1_: Distance to the foreground target, the shutter template
- D_2_: Distance to the background target, the plastic grid template.
- P_o_: Percentage of laser beam blockage by the first target.

So, the distance measured by the sensor was a function of these three variables and could be expressed in the following way:

(1) $$D_{\mathrm{Láser}}=f\;\left(D_{1},\;D_{\mathrm{en}},\;P_{o}\right)$$

where:

- D_Láser_: Distance measured by the sensor
- D_en_: D_2_–D_1_, Distance between the targets.

**Figure 6.**
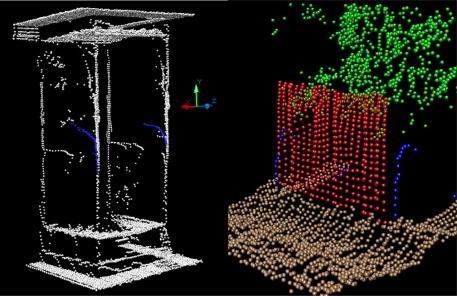
The blue-coloured points are manifestations of the edge effect (mixed pixels) when the laser beam partially fell on the edge of some targets.

In Figure 6 the points which resulted from the edge effect (mixed pixels) when the beam partially fell on some of the targets is shown in blue. The curve described by the blue points reflects the progressive increase or decrease in the percentage of laser beam blockage P_o_ during the movement of the sensor.

**Figure 7.**
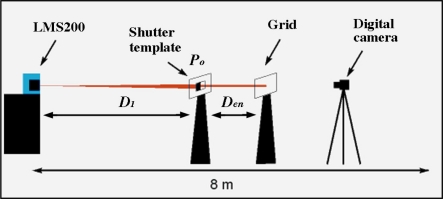
Test set-up to study the function: D_Láser_ = f (D_1_, D_en_, P_o_).

The same test set-up as in the first mixed pixel test was used to study function (1) (Figure 7). The methodology comprised of a series of measurements under different situations, varying the values of D_1_, D_en_ and P_o_. The shutter template (D_1_) was positioned at distances of 1, 2, 3, 5 and 7 m from the sensor. The grid template (D_2_), onto which the unblocked part of the beam was projected, gradually approached D_1_, but in an unsmooth way and in accordance with the previous D_láser_ readings. With respect to the blockage percentage of the foreground target (P_o_), the blockages were performed on the laser beam emitted at 90° using the rectangular template (template D in Figure 5), progressively blocking the beam 5–6 times with horizontal movements. The exact determination of the blockage percentage was obtained from the beam photographs taken at each blockage.

There were a total of 736 different measurement scenarios combining D_1_, D_2_ and P_o_. Data organisation and analysis was performed with version 6.5 of the Matlab software. In each measurement situation D_Láser_ was obtained from the means of 10 consecutive individual measurements. In this way, any small differences between measurements made in ‘equal’ situations were smoothed out.

## 3. Results and Discussion

This section deals with both the results of the study undertaken on the laser beam geometry and the results of the study undertaken on partial blockage of the beam. These results should enable a better understanding of the edge effect (mixed pixels), which is produced when the beam does not fully fall on a single target.

### 3.1. Visualization and Geometric Characterisation of the LMS200 Sensor Laser Beam

**Figure 8.**
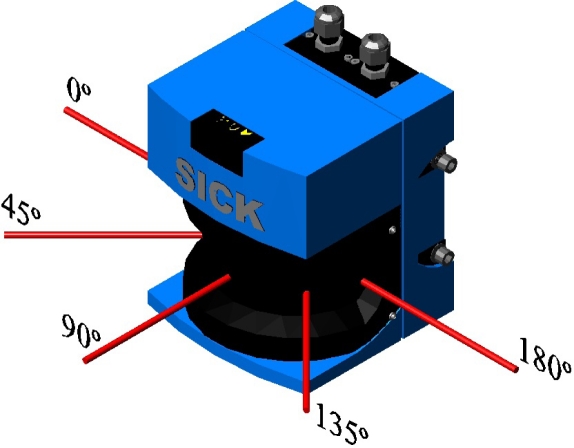
Laser beam paths emitted by the LMS200 sensor at 0°, 45°, 90°, 135° and 180°.

**Figure 9.**
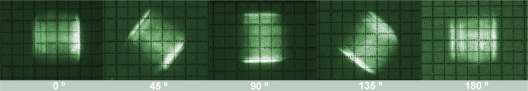
Photograph of the cross-sections of the beams emitted at 0°, 45°, 90°, 135° and 180°.

A first result of laser beam visualisation was that its cross-section was rectangular in shape (Figure 9) and not circular, as indicated by the manufacturer [15]. It was also noted that its intensity profile was not homogenous. Figure 8 shows the laser beam paths emitted at 0°, 45°, 90°, 135° and 180°.

Figure 9 shows a photograph of the cross-section of each of these beams. The beam can be seen turning at the same time as the internal rotating mirror of the sensor in the photograph sequence.

**Figure 10.**
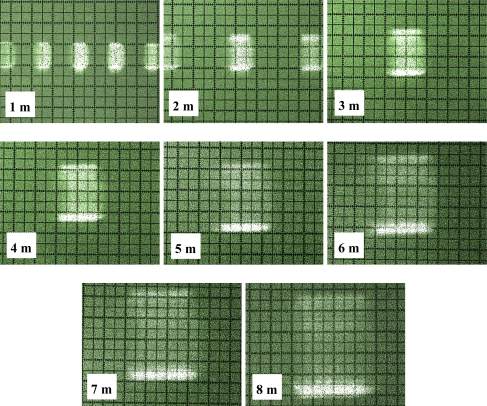
Photographs of the laser beam cross-section at distances of between 1 and 8 m from the sensor. The dimensions are shown in Table 1. The smallest subdivisions were 1 mm.

Figure 10 shows the photographs of the beam cross-sections at distances of 1, 2, 3, 4, 5, 6, 7 and 8 m from the sensor. It can be clearly seen from the photograph sequence how the size of the beam grew with distance due to its divergence.

The manufacturer [16] defines the LMS200 laser emission as a circular shaped beam with an initial output diameter equal to 12 mm and with a divergence of 4.4 mrad. In Table 1, a comparison is made between the information supplied by the manufacturer and the dimensions obtained from beam photographs. It can be seen that the diameter as calculated from the information provided by the manufacturer and the measured beam spot dimensions in the photographs of Figure 10 are very similar. The fast axis direction corresponds to the vertical direction in Figure 10, while the slow axis direction corresponds to the horizontal direction.

Using the photographs, a simplified modelling of the laser beam was carried out as a truncated pyramid shaped figure. This model was created using measurements of cross-sections of the beam.

The beam width, in mm, of the slow-axis (*w*_s_) for any distance (*x*), is obtained from the following expression:

(2) $$w_{s}=0.0037\cdot x+4.74\;(mm)\;R^{2}=1$$

The beam width, in mm, of the fast-axis (*w_f_*) for any distance (*x*), is obtained from the following expression:

(3) $$w_{f}=0.0052\cdot x+6.54\;(mm)\;R^{2}=1$$

From Equations (2) and (3), it can be deduced that the laser beam has a divergence of approximately 3.7 mrad along the slow axis and of 5.2 mrad along the fast axis, rather than a single divergence of 4.4 mrad, as specified by the manufacturer. The laser beam must also have an astigmatism since the position of the beam focuses for the two axes are different, as can be appreciated from Equations (2) and (3).

### 3.2. First Mixed Pixel Test: Partial Blockage of the Laser Beam at a Distance of 5 m

**Figure 11.**
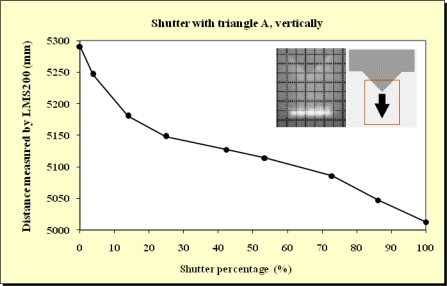
Graphic representation of the evolution of the distance measurement given by the sensor when the laser beam (90°) was incrementally blocked (from top to bottom) using the triangular shaped template A at a distance of 5 m from the sensor and the unblocked part of the beam was captured by the grid template at a distance of 5.3 m.

**Figure 12.**
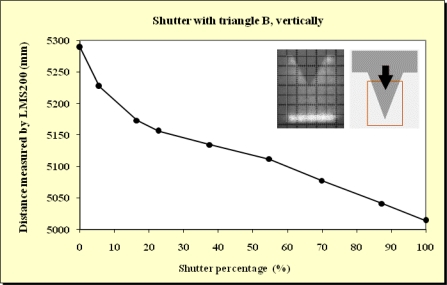
Graphic representation of the evolution of the distance measurement given by the sensor when the laser beam (90°) was incrementally blocked (from top to bottom) using the triangular shaped template B at a distance of 5 m from the sensor and the unblocked part of the beam was captured by the grid template at a distance of 5.3 m.

**Figure 13.**
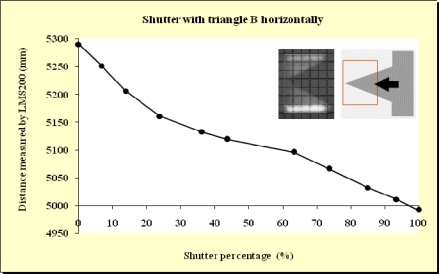
Graphic representation of the evolution of the distance measurement given by the sensor when the laser beam (90°) was incrementally blocked (from right to left) using the triangular shaped template B at a distance of 5 m from the sensor and the unblocked part of the beam was captured by the grid template at a distance of 5.3 m.

**Figure 14.**
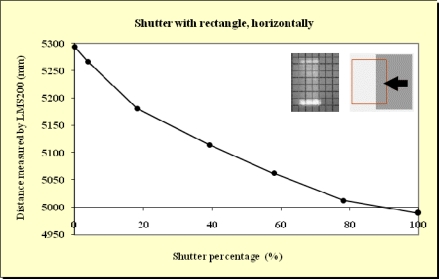
Graphic representation of the evolution of the distance measurement given by the sensor when the laser beam (90°) was incrementally blocked (from right to left) using the rectangular shaped template at a distance of 5 m from the sensor and the unblocked part of the beam was captured by the grid template at a distance of 5.3 m.

**Figure 15.**
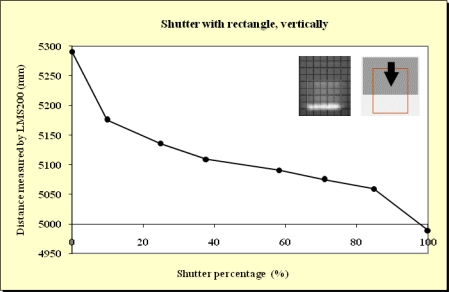
Graphic representation of the evolution of the distance measurement given by the sensor when the laser beam (90°) was incrementally blocked (from top to bottom) using the rectangular shaped template at a distance of 5 m from the sensor and the unblocked part of the beam was captured by the grid template at a distance of 5.3 m.

In this study, beams emitted at 90°, 45° and 0° were blocked. As expected, no differences were observed. This meant that the blockages did not depend on which beam was obstructed. As can be observed from the Figures below (Figures 11–15), when the beam was partially blocked and fell on two elements at different distances the LMS200 sensor attributed intermediate distances to both elements. In Figures 11–13, where the blockages were made using triangular-shaped templates A and B both vertically and horizontally, it can be seen that there was no linear relationship between the shutter percentage and the distance given by the sensor. In Figure 15, looking at the curve which joins the points, it can be clearly seen that the slope falls more sharply at both the start (P_o_ < 10) and end (P_o_ > 85) of the curve than in the central part. These two areas, at the beginning and end of the curve, coincide with the blockage of the two horizontal bands which are notable for their higher irradiance in the upper and lower part of the beam cross-section. This result would appear to indicate that the distance value given by the sensor was more related to the blocked radiant power than to the blocked surface area of the beam.

In Figure 14, the relationship between the shutter percentage and the distance given by the sensor is much more linear than in Figure 15. When considering the manner in which the blockage was effected (rectangular template from right to left), it can be seen that the blockage of the two bands of higher irradiance was made progressively, and not abruptly as in the case of the blockage shown in Figure 15. This provides confirmation that the distance given by the sensor fundamentally depends on the blocked radiant power.

To check and evaluate the influence of irradiance on the range value, the beam was partially blocked in different areas with the 5 mm wide rectangular template [Figure 5(c)] which was moved in a horizontal direction (Table 2). The shutter percentage obtained with this template (P_o_) was 15.43%. The rows in Table 2 are ordered according to the distances measured by the sensor. It can be seen how, while blocking the same beam cross-section percentage (15.43%), the sensor gave different distance values depending on which part of the beam section had been blocked. The first four rows correspond to blockages to the far right and left of the beam emitted at 0°. The last three rows, which gave the highest distance values, corresponded to blockages of the central area of the beam.

The next logical step was to quantify the blocked radiant power in order to relate it to the distance given by the sensor, but there were two problems that made it difficult to carry out this calculation. The first lay in the fact that it is not possible to know how the two previously mentioned internal corrections were carried out [16], based on one table of distances and another of reflectivities, in order to calculate the distance and how these algorithms are affected by partial ray blockage. The second difficulty lay in the photographic camera that was used. In the infra-red vision mode, it did not allow any manual adjustments of the speed of obstruction, nor in the choice of diaphragm or sensitivity. Furthermore, the automatically chosen parameters (obstruction, diaphragm, sensitivity…) were not registered on the photographs either. The fact of having images of obstructed rays produced under different light conditions (with different blockage percentages) automatically implied that the camera parameters were also automatically adjusted in different ways according to each lighting situation, making it impossible to correctly quantify blocked radiant power from the photographs that were taken. Due to these two difficulties, it was not possible continue with this line of work.

### 3.3. Second Mixed Pixel Test: Partial Blockage within the Range from 0 to 8 m

After combining different values for the distance to the first target (D_1_), the distance to the second target (D_2_) and the shutter percentage (P_o_), 736 different measurement scenarios were created and the distance measured by the sensor (D_láser_) was obtained for each of them. The D_1_ values used were 1,000, 2,000, 3,000, 5,000 and 7,000 mm. Table 3 shows some of the distances obtained when the shutter template [Figure 5(d)] was positioned 2,000 mm from the sensor (D_1_ = 2,000 mm).

**Figure 16.**
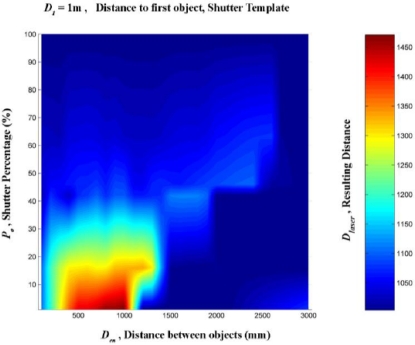
Graphic representation of the distance values (D_láser_) in mm, interpolated from the results of the progressive blockage of the beam at 90° with the shutter template at a distance (D_1_) of 1,000 mm from the sensor, with different values of D_en_ ranging between 0 and 3,000 mm.

**Figure 17.**
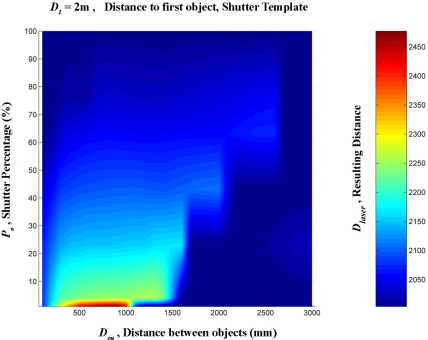
Graphic representation of the distance values (D_láser_) in mm, interpolated from the results of the progressive blockage of the beam at 90° with the shutter template at a distance (D_1_) of 2,000 mm from the sensor, with different values of D_en_ ranging between 0 and 3,000 mm.

**Figure 18.**
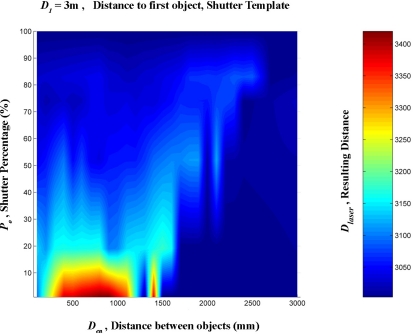
Graphic representation of the distance values (D_láser_) in mm, interpolated from the results of the progressive blockage of the beam at 90° with the shutter template at a distance (D_1_) of 3,000 mm from the sensor, with different values of D_en_ ranging between 0 and 3,000 mm.

**Figure 19.**
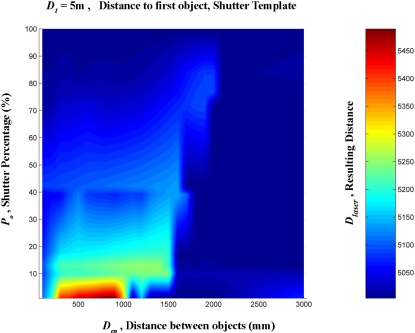
Graphic representation of the distance values (D_láser_) in mm, interpolated from the results of the progressive blockage of the beam at 90° with the shutter template at a distance (D_1_) of 5,000 mm from the sensor, with different values of D_en_ ranging between 0 and 3,000 mm.

**Figure 20.**
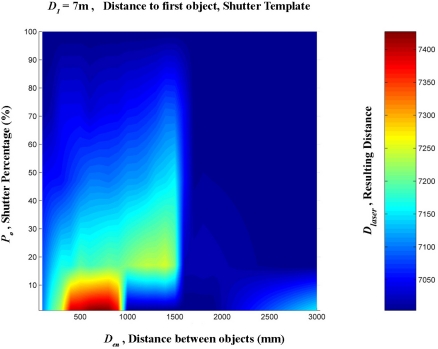
Graphic representation of the distance values (D_láser_) in mm, interpolated from the results of the progressive blockage of the beam at 90° with the shutter template at a distance (D_1_) of 7,000 mm from the sensor, with different values of D_en_ ranging between 0 and 3,000 mm.

A linear interpolation was them made from these data. This was done in order to create a regular mesh in which the shutter percentage increased by one degree at a time, between 0 and 100%, and the distance between the targets increased by 100 mm at a time, from 0 to 3,000 mm. Figures 16–20 provide graphic interpretations of the interpolated distances (D_láser_) with values of D_1_ equal to 1,000, 2,000, 3,000, 5,000 and 7,000 mm.

**Figure 21.**
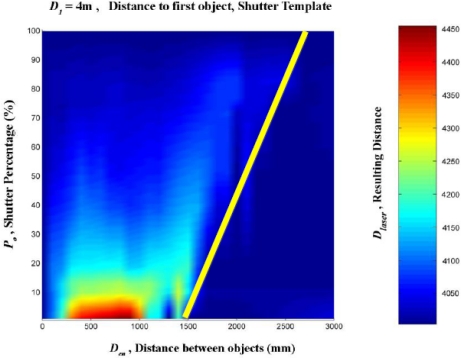
Graphic representation of the interpolated distance values (mm) for the case of D_1_ = 4,000 mm. The left-hand area delimited by the yellow line corresponds to the area of influence of the second target hit by the laser beam.

In Figures 16–20, an area can clearly be seen (of different colours to the dark blue) in which the second background target (D_2_) had an influence on the distance given by the LMS200 (D_láser_) and another dark blue area in which the second target had no influence. This area of influence is bounded, in a simplified way, by an imaginary line (Figure 21) which begins at approximately D_en_ = 1,500 mm when the blockages are small (P_o_ < 30) and which reaches D_en_ values of approximately 2,500 mm when the blockages are large (P_o_ > 60). For a given blockage, when D_en_ is to the left of this imaginary line the second target had an influence on the measurement given by the sensor D_láser_. However, when D_en_ is to the right of the imaginary line, the second target was ignored by the sensor, with D_láser_ being the distance to the first target.

Using the results obtained with D_1_ values of 1,000, 2,000, 3,000, 5,000 and 7,000 mm, the results could be obtained through interpolation of any other distance D_1_, as shown in Figure 21 with D_1_ = 4,000 mm.

As it is possible to observe in Figures 16–20, in the case of an impact on two different objects, with a distance between them, the result of the measurement provided by the LMS200 sensor (D_láser_) depended on the values for the variables D_1_, D_en_ and P_0_, which gave either the distance to the first object or an intermediate distance between the two. We did not obtain any absurd measurements beyond the second object or before the first one. In this respect, the sensor worked excellently and it was therefore not necessary to use any other mechanism to eliminate erroneous measurements. From the previous affirmation, it is possible to deduce that, in this case, the problem of mixed pixels was not considered an error, but rather a technical characteristic of the sensor which should have been explained in the users’ manual.

One application of the geometric characterisation of orchard crops is that of obtaining and measuring the total volume occupied by the trees. To quantify the error present when obtaining a determined volume from a cloud of points provided by a LIDAR sensor, it is first necessary to know what the real volume with which to compare it is. This is where the problem of determining the true volume occupied by a tree arises. One objective way to determine this, which is easy to understand though very difficult or impossible to perform in practice, is to obtain the occupied volume by immersion in a tank of water. In this case, the mesh of points that corresponds to the external surface of the tree volume is practically infinite. As a result, any other mesh of points will always have a lesser resolution and will therefore provide us with a greater tree volume. Depending on how it is measured, a given type of vegetation will occupy one volume or another. The discussion relating to which volume is the best or most appropriate will depend on the subsequent use that we give to this volume. To interpret a volume and relate it to other parameters, such as—surface area or leaf density in the case of vegetable material, it is necessary to know the resolution and process for obtaining the mesh of points and to be aware of the mixed pixel problem. This same affirmation could be equally applied to any other information derived from the cloud of points, such as—the surface area around the vegetation, projected surfaces, roughness, distribution of points, *etc.*

Applications based on LIDAR sensors should, according to their objectives, evaluate and quantify the influence of the mixed pixel problem, even if it is necessary to do this through trials. Within the specific case of the geometric characterisation of tree crops using the LMS200 sensor, the results presented in this work could be useful for making an initial evaluation of the mixed pixel effect. Depending on the objectives of each work, we should not rule out, according to the case, the need to carry out specific trials to quantify the effect of mixed pixels as precisely as possible.

## 4. Conclusions

Visualisation of the beam emitted by the LMS200 sensor is acceptable and reveals characteristics not specified by the manufacturer [15,16]. In terms of the new information that has been acquired, it was observed that the laser beam cross-section is rectangular shaped, that it turns at the same time as the internal rotating sensor mirror and that its irradiance profile is not homogenous, with some areas displaying more radiant power than others.

Using measurements made from photographs of the laser beam cross-section at distances of 1–8 m, an approximate determination was made of its divergence along the slow (3.7 mrad) and fast axes (5.2 mrad), with the additional observation that the beam has an astigmatism.

The diameter of the laser beam, as calculated from the information provided by the manufacturer (circular spot), at distances of 1, 2, 3, 4, 5, 6, 7 and 8 m, approximately coincides with the largest dimension of the rectangular sections of the beam at the same distances. The circular spot defined in the technical specifications therefore circumscribes the actual rectangular section beam emitted.

The conclusion drawn from the first partial blockages of the beam, made at 5 m from the sensor, is that the distance value depends more on the blocked radiant power than on the blocked surface area of the beam.

The conclusion that can be drawn from the second mixed pixel test, in which partial blockages were made between 0 and 8 m, is that there was an area of influence which was dependent on the shutter percentage (P_o_). This varied from 1.5 to 2.5 m with respect to the foreground target, so if the second target impact of the laser beam occurred within that range it would affect the measurement given by the sensor. However, when the second target was outside this area of influence, the sensor ignored this second target and gave the distance to the first impact target.

As a final consideration, in the geometric characterisation of the tree crops, to interpret the information obtained from clouds of points generated by LIDAR sensors, such as—the volume occupied, enclosing surface area, projected surfaces, roughness, distribution of points, it is necessary to know the resolution and the process by which this mesh of points was obtained. This also implies having some knowledge of the problem of mixed pixels, which is perhaps best understood as a technical characteristic of the sensor used and not as an error.

## Figures and Tables

**Table 1.** Dimensions of the LMS200 sensor laser beam according to the laboratory tests and according to the information supplied by the manufacturer [16].

	
	**Dimensions According to Beam Cross-Section Photographs**		**Dimensions According to the Manufacturer**

**Dist. (m)**	**Slow axis (mm)**	**Fast axis (mm)**	**Beam Diameter (mm)**
0	-	-	12.0
1	8.5	11.7	16.4
2	12.2	16.9	20.8
3	16.0	22.1	25.2
4	19.7	27.2	29.6
5	23.4	32.4	34.0
6	27.2	37.6	38.4
7	30.9	42.8	42.8
8	34.7	47.9	47.2

**Table 2.** Distance given by the LMS200 when different parts of the beam were blocked with the 5 mm wide rectangular template at a distance of 5 m from the sensor and when the unblocked part of the beam was captured by the grid template at a distance of 5.3 m.

**Blockage with 5 mm Wide Rectangular Template**					

**Photograph**	**Total surface area (mm^2^)**	**Blocked surface area. (mm^2^)**	**% blockage**	**Distance measured (mm)**	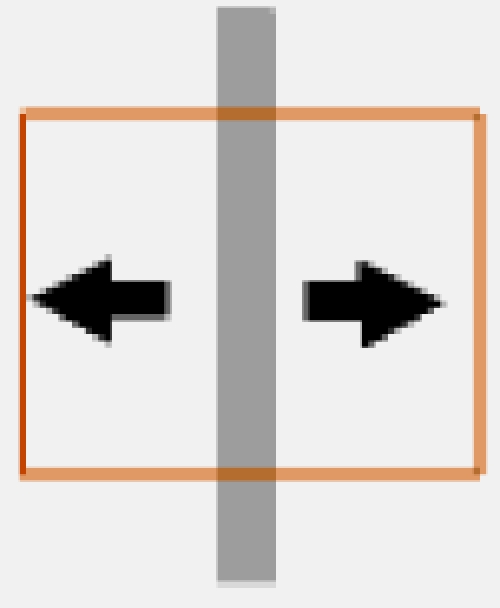
DSC30620	835.33	128.87	15.43	5136.8	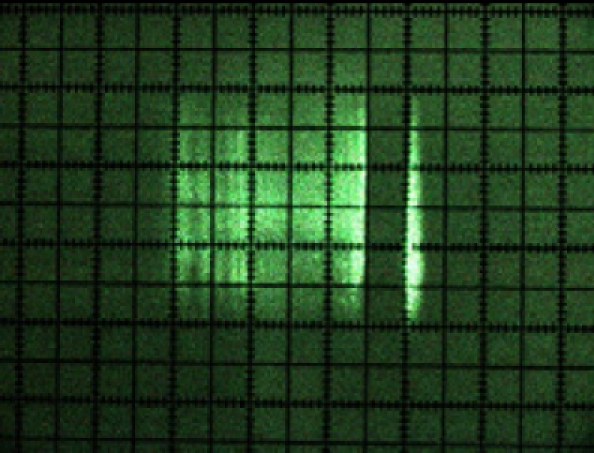
DSC30622	835.33	128.87	15.43	5154.3	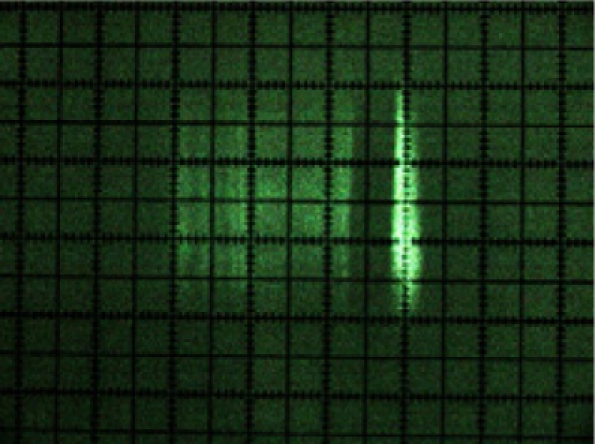
DSC30616	835.33	128.87	15.43	5178.1	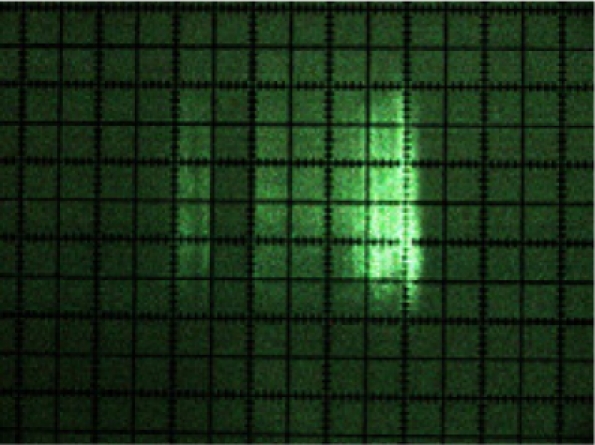
DSC30615	835.33	128.87	15.43	5182.2	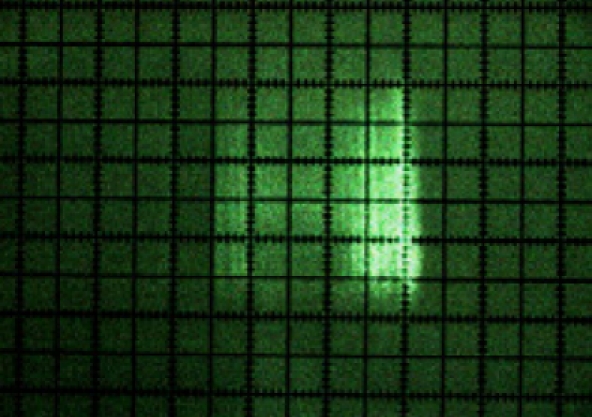
DSC30619	835.33	128.87	15.43	5184.4	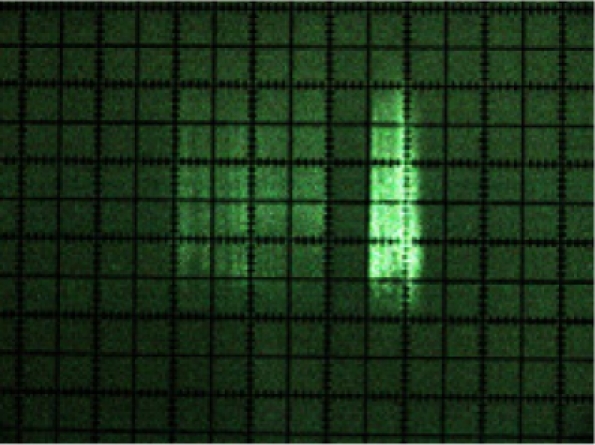
DSC30617	835.33	128.87	15.43	5197.5	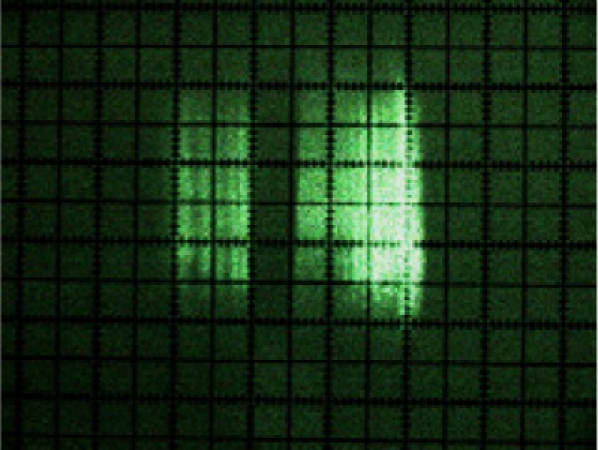
DSC30618	835.33	128.87	15.43	5202.5	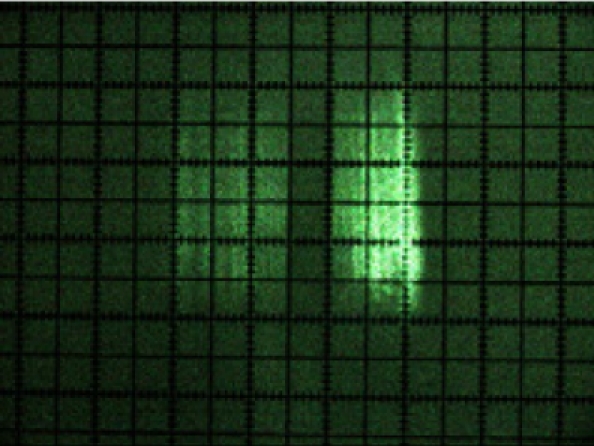

**Table 3.** Distances in mm given by the LMS200 sensor when the beam at 90° was progressively blocked with the shutter template at a distance (D_1_) of 2,000 mm from the sensor, with different values of D_en_ ranging between 0 and 3,000 mm. Only part of the results obtained are shown here.

**Distance between Targets *D_en_*: 0 to 3,000 mm**														

		**20**	**50**	**100**	**200**	**300**	**400**	**500**	**600**	**700**	**800**	**900**	**1,000**	**(…)**
**Shutter percentage, *P_o_***	**1%**	2001	2017	2067	2180	2281	2361	2431	2443	2468		2481	2426	…
	**4%**	1997	2019	2079	2156	2217	2226	2235	2241		2245		2248	…
	**23%**	1997	2017	2066	2104	2135	2146	2146	2151	2150	2149	2152	2154	…
	**43%**	2000	2010	2033	2055	2083		2093			2098			…
	**63%**		1997	2000	2009	2035	2045		2053		2056			…
	**76%**		1989	1990	1996	2009	2016	2012	2016	2032	2034		2031	…

**Shutter template *D_1_*= 2,000 mm**
